# Oral manifestations associated with gastrointestinal diseases - A review

**DOI:** 10.6026/973206300210383

**Published:** 2025-03-31

**Authors:** Aishah Ansari, Sayem Anwarhussain Mulla, Amit Patil, Aarti S. Bedia, Saudamini More, Himmat Jaiswal, Sheetal Mali, Shefali Talekar, Shruti Singh

**Affiliations:** 1 Dental Surgeon, Bharati Vidyapeeth (Deemed to be University) Dental College and Hospital, Navi Mumbai, Maharashtra, India; 2 Department of Conservative Dentistry & Endodontics, Bharati Vidyapeeth (Deemed to be University) Dental College and Hospital, Navi Mumbai, Maharashtra, India; 3 Department of Oral Medicine & Oral Radiology, Bharati Vidyapeeth (Deemed to be University) Dental College and Hospital, Navi Mumbai, Maharashtra, India; 4 Department of Public Health Dentistry, Bharati Vidyapeeth (Deemed to be University) Dental College and Hospital, Navi Mumbai, Maharashtra, India

**Keywords:** Oral manifestations, gastrointestinal disorders, gastrointestinal disorders, gastrointestinal tract (GIT) disorders

## Abstract

Gastrointestinal diseases have become more prevalent in recent years and oral symptoms frequently precede systemic ones. These oral lesions might be the consequence of systemic alterations or have a direct connection to gastrointestinal lesions. Functioning and appearance are impacted by these lesions and it affects both soft and hard oral tissues. Hence, early detection of oral symptoms is essential for the diagnosis of underlying gastrointestinal problems. Thus, the significance of recognizing these oral signs to facilitate timely diagnosis and treatment is emphasized in this review.

## Background:

Recording a thorough medical history of a patient plays an important role in the diagnosis of any dental conditions and the overall health of an individual which also helps in better postoperative care for the patient and certain "incidental findings" may lead to the detection of underlying hidden systemic illnesses. Oral findings are routinely known to precede any other significant systemic manifestation [1]. The oral cavity serves as a gateway to the gastrointestinal tract as it shares an embryogenic origin with it and due to this; many gastrointestinal disorders show characteristic or pathognomonic signs and symptoms in the oral cavity [2]. These oral lesions at times may precede the gastrointestinal lesions. Subsequently, they may remain throughout the disease process or persist even after the gastrointestinal lesion has resolved. Every so often, the oral lesions may mimic the gastrointestinal lesion while otherwise they may be a consequence of systemic alteration brought about by the gastrointestinal disease (*e.g.* mal-absorption) [3]. A novel proposed classification system of gastrointestinal diseases affecting the oral cavity is given in Table 1. Therefore, it is of interest to review various gastrointestinal disorders and their oral manifestations.

## Ulcerative colitis:

Oral manifestations include pyostomatitis vegetans, ulcerations, tongue coating, recurrent aphthous stomatitis (RAS), geographic tongue, atrophic glossitis, fissured tongue, burning mouth syndrome, dry mouth, angular cheilitis, taste changes, acidic taste, halitosis as well as periodontitis [4, 5]. Pyostomatitis vegetans is the medical term for ulcerative colitis oral lesions [6, 7]. When compared to Crohn's disease, it has far less common and rare involvement of the oral cavity. Males are more commonly affected and oral lesions can appear at any age. Oral lesions are thought to come before gastrointestinal lesions, but they are usually synchronous. These lesions may be either clumped or scattered, or linear-oriented pustules resting on an erythematous mucosa, affecting various sites in the oral cavity with varying severity but excluding the dorsum of the tongue. The level of ulceration in the lesions, which may often remain epithelized, is directly proportional to the patient's discomfort. Long-term lesions may become granular, polypoid, or fissured, resembling Crohn's disease. In addition to pustular lesions, some patients may develop an oral aphthous lesion. Approximately 10% of patients are more likely to develop inflammatory bowel disease-related temporomandibular joint (TMJ) arthritis [6].

On microscopic examination, the lesions usually resemble colonic crypt abscesses but do not have any granulomatous inflammation. The oral submucosa shows edema with neutrophils, eosinophils and lymphocytes, whereas the epithelium shows spongiosis in conjugation with neutrophilic and eosinophilic abscesses [7]. The microscopic appearance is not pathognomonic because neutrophilic or eosinophilic intraepithelial abscesses can also be seen in candidiasis, benign migratory glossitis, stomatitis areata migrans and even pemphigus vegetans [8]. The clinical appearance and history must be studied together along with the microscopic findings; otherwise, asymptomatic patients receiving an oral diagnosis of pyostomatitis vegetans should be evaluated for bowel disease by a gastroenterologist [7]. Oral lesions usually respond to treatment for colonic disease. For recalcitrant oral lesions, topical or systemic corticosteroid therapies as well as dapsone have been used with varying degrees of success [6, 7].

## Gastroparesis:

Gastroparesis is termed to be a rare but debilitating gastrointestinal motility disorder often terminating in complete or partial paralysis of the muscles of the stomach. The presence of symptoms, the absence of stomach obstruction and delayed gastric emptying all contribute to the diagnosis of gastroparesis; however, the etiology of this disorder is complex. Postoperative gastroparesis has been linked to the diagnosis of stomach muscle impairments and several underlying health conditions, including Parkinson disease, neurological disorders, scleroderma, post-viral infections and most commonly, diabetes. While there are many known risk factors for the development of gastroparesis, more than half of all cases are idiopathic [9].

When the oral cavity is concerned, there are evident signs of hypo-salivation in severe cases of malnutrition or due to antiemetic drugs. Patients with decreased salivary flow, frequent nausea and vomiting and strict dietary restrictions are more likely to develop erosive tooth wear (ETW), dentinal hypersensitivity and caries. Vomiting, dietary restrictions, early satiety and/or slow gastric emptying rates all contribute to malnutrition, which has a negative impact on periodontal health and the development of oral conditions related to vitamin and nutrient deficiencies, such as glossitis, angular cheilitis, pallor gingiva, stomatitis and oral malodor. Due to this, there are clear signs such as enamel and dentin hypoplasia, decreased saliva, ageusia, burning tongue, angular cheilitis, purple/magenta tongue, scarlet glossitis, ulcerations, gingival inflammation, periodontal diseases, stomatitis, hemorrhagic gingiva, bone loss, oral malodor, delayed wound healing, defective collagen formation, bleeding gingiva, dental caries, abnormal alveolar bone patterns, gingival detachment, pallor lips/mucosa, salivary gland dysfunction, changes in filiform papillae, hyposalivation, *etc.* Other effects include saucer-shaped depressions on the lingual aspects of the teeth due to acid and erosion leading to dental caries and hypersensitivity [9, 10].

## Peptic ulcer and Helicobacter pylori infection:

Peptic ulcer can be either gastric or duodenal. Patients with peptic ulcer are geriartic individuals, smokers or alcohol drinkers [1]. The patients may complain of abdominal pain primarily arising from epigastrium. Constitutionals symptoms include regurgitation, indigestion, nausea, vomiting, chest pain as well as appetite loss [11, 12]. If left untreated, possible sequelae include grievous erosion, which can lead to perforation and/or serious hemorrhage and most have a risk of malignant transformation [13]. *H. pylori* infection is not only connected to peptic ulcers but also to a myriad of other pathological conditions including gastric cancers [14, 15]. *H. pylori* is a class I (definite) human carcinogen as per World Health Organization (WHO) [16]. *H. pylori* present in the oral cavity which is a potential extra-gastric reservoir of it [17, 18-19]. It has been isolated from saliva, tongue and supra as well as sub gingival plaque [20, 21- 22]. It co-aggregates with Fusobacterium species during dental plaques biofilm formation [23]. Further, it is isolated in greater quantities in the plaque biofilm of individuals with peri-implantitis [24].

*H. pylori* of the oral cavity and stomach share a strong positive relationship in their presence [19, 25, 26- 27] although some have claimed its presence to be transient and unrelated to the status of the oral cavity [28, 29-30]. *H. pylori* present in the oral cavity seem to be a potential source of re-infection [31]. Patients suffering from chronic atrophic gastritis caused by *H. pylori* depict significant periodontal attachment loss [32]. Antibiotic therapy alone cannot suffice the eradication of *H. pylori* [18]. But periodontal therapy as an adjunct helps in reducing the gastric content of it significantly [33, 34- 35].

## Constipation:

Common conditions are affecting virtually all age groups and genders. Characteristic symptoms and signs include fewer bowel movements than normal, pain and straining when passing stools, stomach pain along with hard and dry stools. Oral signs and symptoms; although not characteristic are bad taste in the mouth, bad breath, *etc.*.

## Crohn's disease:

Mostly affecting the ileocolonic region of the lower gastrointestinal tract, Crohn's disease is a chronic inflammatory bowel illness. Crohn's disease affects men and women equally and has a bimodal age distribution associated with its onset, with two peaks: one between the ages of 20 and 40 and the other between 50 and 60. Although of unknown etiology, Crohn's disease has a strong genetic correlation and is linked to a changed immunological response. In addition, any change in the commensal microbiota of the gut due to smoking, drug use, modified diet or infectious processes, has also been linked to the onset of Crohn's disease. Crohn's disease frequently manifests in the oral cavity and these symptoms may be the first to show. Aphthous ulcers, edema, pain and redness are among the initial symptoms. They are mostly found on the tongue, lips and mucosa. Both specific and non-specific manifestations exist. The granulomatous substrate and non-caseating inflammation, mainly present in specific oral manifestations, are what distinguish each of these. Miescher cheilitis or lips with granulomatous changes and ulcers are examples of specific presentations. On the buccal mucosa, indurated polypoid tumors can occasionally be seen. Erythema nodosum, oral aphthous ulcers and various other neutrophilic dermatoses are a few examples of non-specific presentations. Oral clinical manifestations act as early indicators of the disease and are crucial in the detection and prompt diagnosis of the disease. As a result, the patient's prognosis and quality of life both improve [37].

## Celiac disease:

It is an autoimmune disease showing genetic predisposition, characterized by an inflammatory reaction in the intestinal villi due to the ingestion of gluten containing food. Abnormalities in the structure of the dental enamel, including hypoplasia and hypomineralization, have been documented in children diagnosed with Celiac disease. Defects like pitting, grooving or complete loss of enamel may also occur. Recurrent aphthous ulcers and cheilosis have also been commonly documented in patients of celiac disease [37]. The enamel defects in celiac disease have been classified into four grades by Aine and colleagues [39].

Grade 1: Defects in the color of enamel, such as single or multiple cream, yellow or brown opacities

Grade 2: Minute structural defects, including rough enamel surface, horizontal grooves and shallow pits

Grade 3: Evident structural defects like deep horizontal grooves and large pits

Grade 4: Severe structural defects, involving a change in the shape of the tooth

## Gardner syndrome:

This autosomal dominant (or sporadic mutation) disease is caused by a chromosomal 5 genetic abnormality and is characterized by intestinal polyposis that carries a very high risk of developing into colonic adenocarcinoma [39]. Numerous extracolonic alterations that impact multiple organ systems, including the skin, bone and soft tissues have been associated with it [8]. Potential head, face and neck symptoms that typically appear in childhood or adolescence include [40].

[1] Multiple enostoses of the maxilla and mandible

[2] Supernumerary and/or unerupted teeth

[3] Higher risk of odontomas

[4] Osteomas of the paranasal sinuses and jaws

[5] Epidermoid cysts of the head and neck

Enostosis frequently presents with no symptoms at all. Radiographs of the alveolar regions of the jaws often show enostoses without any indication of bone expansion. The incisor, cuspid and bicuspid regions are the most frequently affected by supernumerary and unerupted teeth, with the molar parts rarely impacted. The majority of supernumerary teeth are misshapen or peg-shaped. Odontomas are compound teeth that resemble supernumerary teeth in their pattern of occurrence. The osteomas can become large enough to be clinically evident and can be felt via the skin or oral mucosa. They induce a focal expansion of the surface of the jaw bone [40].

If the epidermis cysts or osteomas are large enough to cause functional or cosmetic issues, they are surgically removed. Impacted teeth that are asymptomatic may be kept in the jaws if there is no clinical reason to extract them. However, in some cases, one or more of these additional teeth may need to be extracted for orthodontic and/or occlusion reasons, as well as for cosmetic reasons. Odontomas are usually surgically curetted. To help diagnose the illness clinically and early in life, the gastroenterologist may utilize the oral symptoms. This will enable adequate screening for adenocarcinoma and intestinal polyposis. According to Ida *et al.* Gardner syndrome should be discussed with patients who have three to six osteomatous lesions of the jaw. If more than six lesions are present, the patient should be presumed to have Gardner syndrome until evidence to the contrary is shown [40].

## Peutz-Jeghers syndrome:

There is a mutation in the LKB1 gene in patients of Peutz-Jeghers syndrome [8]. This condition is either the product of spontaneous mutation or autosomal dominant inheritance and is linked to hamartomatous polyposis [39]. Orally, the most obvious variation is perioral and/or oral pigmentation, which first manifests in childhood [41, 42]. A common feature is non-sun-dependent freckling of the skin around the lips and the vermilion zone of the lips [42]. Intra-orally, the lesions are usually pigmented brown, flat, painless patches of the tongue, buccal or labial mucosa [8, 42]. Microscopically, these lesions show signs of mild acanthosis, rete peg elongation and enhanced pigmentation in the nearby keratinocytes and melanocytes. The number of melanocytes remains unchanged. For pigmented lesions, treatment is not required except in cases with cosmetic or social justification. Potassium-titanyl-phosphate laser ablation showed encouraging results, according to Zaheri *et al.* [41]. Similar to Gardner syndrome, oral manifestations play a crucial role in early diagnosis and enable adequate screening for neoplasms and bowel disorders.

## Achalasia:

Achalasia is an uncommon disease marked by the deterioration of nerve fibers in the esophageal Auerbach's plexus. Consequently, the primary characteristics of the illness are non-peristaltic contractions and the subsequent inability of the esophageal musculature to relax after swallowing. Patients with achalasia often exhibit oral symptoms because esophageal tissues are directly related to orofacial anatomical features. In addition to dysphagia of solids and liquids after swallowing, dental erosion is a long-term manifestation, defined as the loss of hard dental tissue as a result of the pH of the regurgitated acid [43]. In contrast to reflux illness, where the stomach's hydrochloric acid is the cause of acid reflux, the occurrence of palatal dental erosion in achalasia patients strongly supports that lactic acid is the source of the acid in the esophagus [44]. Tooth erosion can be avoided in large part by receiving an early diagnosis of achalasia. Depending on how severe the dental manifestations are, there may be functional and cosmetic issues that require surgical or prosthetic treatments. Once the patients' symptoms have subsided, prosthetic rehabilitation may be necessary depending on the extent of erosion [45].

## Colorectal cancer:

The third most prevalent cancer in the world is colorectal cancer. The clinical symptoms include abdominal pain, alteration of bowel habits and movements, involuntary loss of weight, nausea, vomiting, anorexia, malaise, *etc.* Distal cancers have also been documented to cause rectal bleeding, which can cause mixed blood with stool [46]. Due to a lack of existing literature, the relationship between dento-osseous anomalies in patients with colorectal cancer cannot be clearly established [47]. However, oral ectopic sebaceous glands, also known as Fordyce granules and an alteration in vascular pattern in the oral mucosa are reported changes in oral manifestations of colorectal cancer [48].

## Plummer-vinson syndrome:

Plummer-Vinson syndrome is a very rare, anemia associated disorder. Patients frequently complain of dysphagia, burning sensation in the mouth along with glossitis [49]. Careful management of these patients is of utmost significance due to the high risk of esophageal and pharyngeal carcinomas [49, 50].

## Diverticular disease:

Among the most prevalent gastroenterological conditions is diverticular disease. Its prevalence is linked to a diet that is generally low in fiber, though research on the subject is not entirely conclusive. Additional risk factors include immunosuppression, smoking, alcoholism, obesity, poly-cystosis and hypertension, using opiates, corticoids and non-steroidal anti-inflammatory drugs [51]. Diverticulosis is not harmful in and of itself and the majority of those who have it do not exhibit any symptoms. However, some individuals may observe blood in their stool, suffer unexplainable cramping or pain in their abdomen, or encounter changes in their bowel habits [52]. One study recorded a number of unusual oral manifestations, which helped in the diagnosis of Zenker's diverticulum in a patient. Zenker's diverticulum is an uncommon ailment that affects the upper gastrointestinal system. It is commonly detected within the upper esophageal sphincter, which separates the esophagus from the lower pharynx, on the posterior pharyngeal wall. The most typical symptoms of the condition include dysphagia, coughing, regurgitation, loud liquid swallowing and hoarseness of voice.

On examination of the oral cavity, researchers noted presence of ulcerative lesions on both sides of the buccal mucosa, tongue's dorsal surface and lower lip of the patient. After months of inconclusive oral antibiotic, anti-inflammatory and oral antifungal drug therapy, the patient was referred to a gastroenterologist, for additional assessment. A barium swallow test was advised and based on the radiographic findings the condition was diagnosed as Zenker's diverticulum [53].

## Familial adenomatous polyposis:

Patients with familial adenomatous polyposis (FAP) develop several premalignant colorectal adenomas. If left untreated, one or more of these polyps will develop into colorectal carcinoma in middle-aged adults. Extra-intestinal manifestations of FAP are common and this combination has been dubbed Gardner's syndrome. FAP symptoms in the oral and maxillofacial regions include an increased risk of jaw osteomas, odontomas and supernumerary or unerupted teeth. Early detection of FAP is critical and may save lives. Because oral signs typically precede gastrointestinal symptoms, the dentist may play an important role in the diagnosis of FAP [54].

## Cancers:

Hereditary non-polyposis colorectal cancer (HNPCC) shows presence of dento-osseous anomalies along with Fordyce's granules as an oral manifestation which occurs in a hereditary pattern [47]. Colon cancer metastasizes to the mandible and gingiva more than anywhere else in the oral cavity, accounting for 25% of all oral metastases. There is occurrence of metastasis of colon adenocarcinoma of the maxillary gingiva and palate leading to symptoms such as gingival bleeding, ulcerations and abscesses [55, 56].

## Gastro-esophageal reflux disease:

Gastro-esophageal reflux disease (GERD) is a common digestive disorder linked to substantial morbidity and a lower quality of life. Patients can be identified by both classic and atypical symptoms. The symptoms and complications result from the reflux of stomach contents into the esophagus. A disorder of the lower esophageal sphincter (LES) is the primary cause of gastroesophageal reflux disease; however, a number of other physiological and pathological factors may also play a role in its onset. The most commonly characterized oral manifestation linked to gastro-esophageal reflux disease (and other sources of stomach contents reaching the mouth) is tooth erosion, which has been extensively examined and published in dental literature. Salivary secretions must be adequate to preserve the teeth, as well as the oropharyngeal and esophageal mucosa. Other oral manifestations of gastro-esophageal reflux disease are burning mucosal sensation, halitosis and mucosal erythema. It should be noted that a loss of tooth substance is usually only readily visible after a lengthy time of endogenous acid contact and so, early indicators of erosion may be easily ignored. Aside from tooth erosion, the surfaces of ceramic dental restorative materials that contain a matrix of glass particles and glass-ionomer cement can also be affected to variable degrees by acids. Individuals with gastro-esophageal reflux disease may complain of a sour or acidic taste, poor taste (dysgeusia), an oral burning sensation and water brash (saliva floods the mouth in response to esophageal reflux stimulation).

The potential for tooth erosion is modified by factors such as the composition and pH of the refluxate, the frequency and form it reaches the mouth, the flow rate and buffering capacity of stimulated saliva and whether patients brush softened tooth surfaces immediately after regurgitation episodes. The content and pH of the refluxate, the frequency and manner in which it enters the mouth, the flow rate and buffering capacity of stimulated saliva and whether patients clean softened tooth surfaces promptly following regurgitation episodes all influence the risk of dental erosion [57]. Characteristic features of affected enamel include smoothly glazed appearance with rounded surfaces, enamel thinning leading to increased incisal and proximal translucency and yellowish appearance of the teeth. Active erosion leads to the exposed dentin becoming more sensitive to temperature changes and touch. Exogenous acid sources and superimposed mechanical wear mechanisms (referred to as "erosive tooth wear") can accelerate tooth erosion [58].

## Limitations and future prospects:

Although we attempted to cover almost all gastrointestinal tract diseases in this review, there are gaps for a plethora of diseases that are either unstudied in the current literature or do not exhibit any characteristic oral signs and symptoms that could have aided in their timely diagnosis. Future research is critical for studying and analyzing various gastrointestinal disorders and their associated oral manifestations.

## Conclusion:

It is true that the oral cavity is a window into a person's overall health. Hence, dentists thus play an essential role in the diagnosis of both alarming and non-alarming gastrointestinal disorders. Thus, dentists can help prevent a wide range of systemic illnesses, including gastrointestinal disorders.

## Figures and Tables

**Table 1:** Proposed classification of gastrointestinal diseases affecting the oral cavity

**Genetic**	**Inflammatory**	**Infectious**	**Hypersensitivity**	**Others**
1. Gardner's disease	1. Crohn's disease	1. Peptic ulcer disease	1. Celiac disease	1. Gastroesophageal reflux disease (GERD)
2. Peutz-Jeghers syndrome	2. Ulcerative colitis	2. Helicobacter pylori associated gastritis		2. Pernicious anemia
				3. Plummer Vinson syndrome
				4. Hiatal hernia
				5. Constipation
				6. Gastroparesis
				7. Achalasia
				8. Colorectal cancer
